# A dataset of daily ambulatory psychological and physiological recording for emotion research

**DOI:** 10.1038/s41597-021-00945-4

**Published:** 2021-06-28

**Authors:** Xinyu Shui, Mi Zhang, Zhuoran Li, Xin Hu, Fei Wang, Dan Zhang

**Affiliations:** 1 Department of Psychology, School of Social Sciences, Tsinghua University, Beijing, China; 2 Tsinghua Laboratory of Brain and Intelligence, Tsinghua University, Beijing, China

**Keywords:** Quality of life, Cardiovascular biology, Human behaviour

## Abstract

To better understand the psychological and physiological basis of human emotion, increasing interest has been drawn towards ambulatory recordings of emotion-related data beyond the laboratories. By employing smartphones-based ambulatory assessment and wrist-worn physiological recording devices, the Daily Ambulatory Psychological and Physiological recording for Emotion Research (DAPPER) dataset provides momentary self-reports and physiological data of people’s emotional experiences in their daily life. The dataset consists of ambulatory psychological recordings from 142 participants and physiological recordings from 88 of them over five days. Both the experience sampling method (ESM) and the day reconstruction method (DRM) were employed to have a comprehensive description of the participants’ daily emotional experiences. Heart rate, galvanic skin response, and three-axis acceleration were recorded during the day time. By including multiple types of physiological and self-report data at a scale of five days with 100+ participants, the present dataset is expected to promote emotion researches in real-life, daily settings.

## Abstract (summary)

**Table:** 

Measurement(s)	Heart Rate • Galvanic Skin Response • Emotion
Technology Type(s)	photoplethysmography • Surface Electrode • Day Reconstruction Method • Experience Sampling Method
Sample Characteristic - Organism	Homo sapiens

Machine-accessible metadata file describing the reported data: 10.6084/m9.figshare.13803185

## Background & Summary

The physiological basis of emotion has long been an important research topic in psychology, cognitive neuroscience, and many other fields. This topic has been extensively studied in laboratory scenarios. A common practice is to use standard emotional stimuli such as pictures, sound, text, and video to evoke specific emotions^1–5^ or to ask people to perform certain tasks to elicit emotions such as anger, stress, anxiety^6–10^. The physiological correlations of different kinds of emotions could then be inferred by analyzing the physiological signals during different emotional states. To this end, a variety of datasets have been developed, such as DEAP^11^, MAHNOB-HCI^12^, SEED^13^, DREAMER^14^, AIMGOS^15^, CASE^16^, etc., and heart rate, galvanic skin response (GSR), respiration rate, facial EMG, EEG are among the most commonly recorded physiological signals. These databases are mainly based on laboratory scenarios and have provided important support for the study of the physiological basis of emotions and their applications.

Although these laboratory-based methods allow for strict experimental control, their ecological validity, or the generalizability in real-life scenarios, has been questioned^17–19^. For example, people’s stress intensity, as indicated by their heart rate responses, has been reported to show significantly higher task-related peaks in real-life scenarios such as watching a soccer game than in laboratory scenarios such as a computer math task^19,20^. Besides, human emotions are dynamic, and the dynamic patterns of emotions over hours or days are considered to have significant psychological implications^21–23^. However, the laboratory-based methods were not initially designed to monitor people’s long-term emotional fluctuations in daily life, and it could be difficult to track people’s daily emotional dynamics given the limited time window in most laboratory experiments.

As an essential complement to the laboratory-based methods, the ambulatory assessment methods were proposed to assess people’s momentary states in their daily settings^24–26^. With the popularity of smartphones and the development of wearable sensing devices, this approach has gained increasing attention in recent years^27–29^. The data of ambulatory assessment could include momentary self-reports, physiological data, and observed behaviors. While momentary self-reports can be used to capture people’s emotional experiences in real-time, the corresponding physiological data collected by wearable bio-sensing devices can provide information about their physiological responses in real-life scenarios. Accordingly, a number of physiological studies have demonstrated the potential of the ambulatory paradigm for emotional research, mainly in the clinical and occupational fields^30–35^. In these studies, heart rate and GSR levels have been suggested as the most effective measures for emotion discrimination^19^. More importantly, the ambulatory paradigm can provide rich within-person data, allowing for idiographic analysis that was usually neglected in laboratory experiments. Specifically, recordings over prolonged periods could cover sufficiently diverse scenarios to characterize people’s dynamic patterns of emotions in their daily life^36–38^. However, to the best of our knowledge, no publicly available ambulatory physiological dataset for emotion research has been published.

Here we present a dataset of Daily Ambulatory Psychological and Physiological recording for Emotion Research (DAPPER)^39^. A group of 142 participants was recruited to have a five-day continuous assessment of their emotional experiences, with 88 of them recording physiological signals by wearing a wristband. Heart rate, galvanic skin response as well as three-axis acceleration data were recorded during day time over five days. One critical and challenging issue for studying the physiological aspect of emotion is to annotate the physiological data effectively. Here two most typical ambulatory assessment methods were used to record the participants’ real-time emotional experiences, i.e., the experience sampling method (ESM)^25^ and the day reconstruction method (DRM)^40^. Specifically, the ESM method was operationalized at six random times per day, by asking the participants to report their momentary emotional states on their smartphone; the DRM method was implemented at the end of each day, by asking the participants to recall their day as a continuous series of behavioral episodes in a temporal order and report their emotional states for at least six major events per day. While ESM and DRM have been considered to have similar effectiveness for assessing real-time emotional states^41–43^, having both measurements at the same time is becoming a popular practice in recent years and is believed to provide a more comprehensive description of the participants’ momentary emotional states. In summary, by including multiple types of physiological and self-report data at the scale of five days with 100+ participants, the present dataset is expected to promote researches on the physiological basis of emotion in real-life, daily settings.

## Methods

### Participants

One hundred and forty-eight Chinese volunteers participated in this experiment. Among them, three participants dropped out due to schedule conflict and another one due to device failure. Two participants didn’t give their data-sharing permission. Data from 142 participants (78 females and 64 males, average age 21.5 years, ranging from 18 to 31 years) were retained for the DAPPER dataset.

### Ethical approval

This study was conducted following the Declaration of Helsinki and its later amendments, and the protocol was approved by the local ethics committee of the Department of Psychology, Tsinghua University (No. THU201906).

All participants gave written informed consent prior to their participation. They were informed that the experimental results might be published in academic journals, books or used in teaching.

The permission to share the participants’ raw data records were obtained after the completion of the study with an additional consent form, in which the participants were explicitly informed about the types of data to be shared as well as the potential re-identification risk by sharing the date and time of the raw data records. All participants except two gave their permission.

### Ambulatory psychological data recording

The participants’ psychological data were collected on their mobile phones through the Psychorus questionnaire application (Psychorus, HuiXin, China), using both the ESM and DRM methods. The Psychorus application sent the ESM and DRM questionnaire notifications to the participants’ phones with sound and vibration alerts.

Each ESM questionnaire consisted of 20 items. The first three items collected basic information about daily events. Then, the fourth item asked to what extent do participant shows his/her true self. Next, a five-item TIPI-C inventory^44,45^ was used for the self-assessment of state personality, followed by a ten-item Positive and Negative Affect Schedule (PANAS)^46^, as well as emotional valence and arousal. The selected ten items were upset, hostile, alert, ashamed, inspired, nervous, determined, attentive, afraid, and active. Each questionnaire item was associated with a 5-point scale. The assessments of the true self and state personality were included to provide a complete overview of the participants’ psychological state.

Each DRM questionnaire consisted of a flexible number of basic questionnaire modules. Each module consisted of the same 12 emotional measurement items as the ESM questionnaire, with one additional item that requires the participants to openly describe the event information in a text-based manner (in Chinese). The participants were required to recall their daily activities and report at least six major events (i.e., completing at least six modules) before submitting one DRM report. Once completed, the ESM and DRM reports were uploaded to the Psychorus’s cloud server for storage.

### Ambulatory physiological data recordings

A custom-designed wristband (Psychorus, HuiXin, Beijing, China) was used to record the participants’ physiological data of heart rate and galvanic skin response (GSR) in daily settings^32,47^. Heart rate (HR) was measured by the photoplethysmography (PPG) method: Green light of 532 nm wavelength was used, and the reflected light intensity was measured at a sampling rate of 20 Hz. GSR was measured at the wrist by surface electrodes with conductive gels at a sampling rate of 40 Hz with a resolution of 0.01μS. Three-axis acceleration was recorded at 20 Hz, with a precision of 1/2048 g (the gravity acceleration unit). The raw data were saved to the wristband’s flash memory and exported to the Psychorus’s cloud server every 2–3 days.

### Procedure

**Fig. 1:**
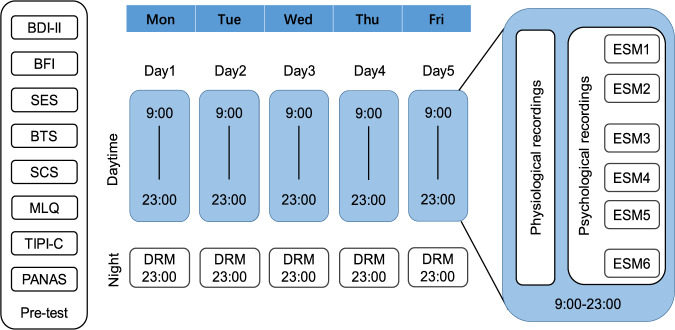
Scheme for daily ambulatory psychological and physiological recordings.

Ambulatory psychological and physiological data were collected from each participant for five consecutive working days (from Monday to Friday) in their daily environment. The experiment was conducted in three batches, each with 28, 56, 58 participants. The dates of the three batches were all during wintertime in 2019, each for five working days. Before starting the data collection, the participants were trained on the data collection procedures, and necessary software was installed on their mobile phones and computers. They were also required to participate in a pre-test and then participated in the formal data collection procedure. The scheme for daily ambulatory psychological and physiological recordings is illustrated in Fig. 1.

#### Pre-test

The following scales (Chinese version) were used in the pre-test session to assess the participants’ basic psychological traits: (1) the Big-Five Inventory (BFI)^48,49^ to evaluate personality traits, with 44 items; (2) the Beck Depression Inventory-II (BDI-II)^50^ to measure the depression level by self-report assessment, with 21 items about situated feelings in the recent two weeks; (3) the Self-Esteem Scale (SES)^51^ to measure self-esteem levels, with 10 items; (4) the Belief in True Selves (BTS)^52^ scale to obtain ontological beliefs about the true self, with 16 items; (5) the Self-Concept Scale (SCS)^53^ to measure independent and interdependent self-construal, with 30 items; (6) the Meaning in Life Questionnaire (MLQ)^54^, with 10 items; (7) the simplified Ten-Item Personality Inventory in China (TIPI-C)^44,45^ for trait measurement, with 5 items; repeated twice for the self-assessment of the general self and the true self; 8) the Positive and Negative Affect Scale (PANAS)^55^ to measure the participants’ predicted emotional experiences in the upcoming week, with ten items. All these scales were conducted online by using the WJX online survey platform (wjx.cn, China).

#### ESM data collectionPre-test

The Psychorus application sent six ESM questionnaire notifications to the participants’ phones every day at random time points between 9:00 a.m. and 11:00 p.m., with a minimal interval of 90 minutes. Upon receiving the notification, the participants were instructed to complete a questionnaire to report their momentary psychological states related to the activities over the past 30 minutes (relative time to start answering the questionnaires). Participants were required to answer all questionnaires as soon as possible after each notification. In cases where participants did not immediately complete the ESM questionnaire after receiving the notification, the system would remind them every five minutes until one hour had passed, after which the push notification would disappear, and the questionnaire would close. The experience sampling of six times per day was decided for the following considerations: (1) six times per day, corresponding to a <3-hour spacing, is believed to capture gross changes in emotional states across the waking day^56,57^; (2) six times per day was confirmed to be an acceptable task load by prior interviews with the participants. During the data collection period, the participants were asked to complete the questionnaires sent by the Psychorus application as many as possible, and they were explicitly informed that a completion rate of >80% was the minimal requirement for a valid data sample.

#### DRM data collection

The participants received one DRM questionnaire notification on 11:00 p.m. every night. They were asked to recall at least six (and no more than nine) most impressive events per day. For every event, they need to give a brief description (in Chinese), report their estimates on the starting and ending time, and perform a full report on their psychological states related to the event.

For the all the psychological data collection including the pre-test, the ESM and the DRM, the participants were presented with all the questions with the default position of the rating bar at the median value of the range (i.e., a default bar position at 4 for a scale with a range from 1 to 7). The participants were required to move the rating bar at least once before submitting their report. This procedure was designed to avoid the participants’ possible mistake of submitting the default value. If the participants intended to choose the median value, they need to move the bar away from the default position and move back.

#### Physiological data collection

In each batch, 28–30 out of the participants were randomly selected to wear the wristbands (28 in the first batch). They were asked to keep the wristbands on as long as possible from 9:00 a.m. to 11:00 p.m., and could only take them off on special occasions such as bathing, swimming, etc. They wore the wristbands on either hand at their convenience, charged the bands during their sleep time, and export data every 2–3 days.

## Data Records

The participant-level information was preprocessed to achieve de-identification in accordance with the Health Insurance Portability and Accountability Act (HIPAA) Privacy Rule^58^ and the guidance for sharing raw clinical data^59^. The inclusion of time and date may help researchers to further investigate the possible influence of weather, air pollution, etc., on human emotion^60,61^. Therefore, time and date information were shared after we acquired permission from all participants.

At the root path of the dataset, it was organized into the following four main directories: (i) Pre-test, (ii) Psychol_Rec, (iii) Physiol_Rec, and (iv) Scripts. The README.TXT files in these directories will give detailed explanations.

For each participant, a unique four-digit participant ID was assigned, with the first digit expresses the batch number.

### Pre-test

This directory contains the results of all the pre-test measurements, which have been integrated into the TRAIT.XLSX file. Each row contains the results from one participant. The organization of the contents in each row is shown in Table 1.

**Table 1:** Organization of the contents for pre-test.

Column	Content	Value range	
1	Submission time	In the format of yyyy/mm/dd HH:MM:SS	
2	Time to complete all questionnaires (in second)	—	—
3	Participant ID	—	—
4–24	BDI-II	1	4
25–29	TIPI-C (according to the general self)	1 (Strongly disagree)	7 (Strongly agree)
30–34	TIPI-C (according to the true self)	1 (Strongly disagree)	7 (Strongly agree)
35–44	PANAS	1 (Not at all)	5 (Extremely)
45–54	SES	1 (Strongly disagree)	4 (Strongly agree)
55–70	BTS	1 (Strongly disagree)	7 (Strongly agree)
71–100	SCS	1 (Strongly disagree)	7 (Strongly agree)
101–144	BFI	1 (Strongly disagree)	5 (Strongly agree)
145–154	MLQ	1 (Strongly disagree)	7 (Strongly agree)

### Psychol_Rec

This directory contains the original records of the DRM and the ESM assessments, as two XLSX files.

#### ESM.XLSX

Each row contains one ESM record (one ESM event) and organized by the participant ID and the start time for answering the ESM questionnaires. The organization of the contents in each row is shown in Table 2.

**Table 2:** Organization of the contents for ESM.

Column		Content	Value range	
1		Participant ID	—	—
2		Start time	—	—
3		Timestamp of start time	—	—
4		Time to complete all items on ESM Page 1 (in millisecond)	—	—
5		Time to complete all items on ESM Page 2 (in millisecond)	—	—
Page 1	6	Place of the event^a^	1	11
	7	Participating people of the event^b^	1	5
	8	Activity type of the event^c^	1	4
	9	The true-self degree	1 (Not at all)	7 (Extremely)
	10–14	TIPI-C for state personality	1 (Strongly disagree)	7 (Strongly agree)
	15–24	PANAS	1 (Not at all)	5 (Extremely)
Page 2	25	Valence	1 (Extremely negative)	5 (Extremely positive)
	26	Arousal	1 (Extremely calm)	5 (Extremely excited)

Note: ^a^Single choice options for place of the event (‘place’) were: classroom, library, student dormitory, playground, gymnasium, canteen, department building (‘department’), other places on-campus (‘on-campus’), home, internship places off-campus (‘internship’), other places off-campus (‘off-campus’);

^b^Multiple choice options for participating people of the event (‘participating people’) were: self, teacher, classmate, family member, and unknown stranger;

^c^Single choice options for activity type of the event (‘activity type’) were: major-related academic activities (‘major’), personal interest-related academic activities (‘interest’), non-academic group activities (‘group’), non-academic personal activities (‘personal’).

#### DRM.XLSX

Each row contains the information about one DRM event (6–9 events per DRM record). The organization of the contents in each row is shown in Table 3.

**Table 3:** Organization of the contents for DRM^a^.

Column	Content	Value range	
1	Event ID	—	—
2	Participant ID	—	—
3	Submission time	—	—
4	Time to complete all DRM items (in millisecond)	—	—
5	The start and end time of the event	In the format of HH:MM	
6–15	PANAS	1 (Not at all)	5 (Extremely)
16	Valence	1 (Extremely negative)	5 (Extremely positive)
17	Arousal	1 (Extremely calm)	5 (Extremely excited)

Note: ^a^The text-based event description was recorded, but was not included in this database to prevent re-identification risks.

### Physiol_Rec

This directory is further separated into 88 sub-folders, and each folder contains all physiological data of one participant. These sub-folders are named by Participant ID, including several CSV files. The preprocessed data were saved in the file named [starting time of the recording]_[ending time of the recording] (referred to as [Physiol_Rec] in the following texts). In this data file, all data were formatted at a 1-Hz sampling rate for the convenience of further analysis. Specifically, the heart rate was computed at a 1-Hz sampling rate with PPG data from a preceding 10-sec time window. The computation was implemented in the software package provided by the manufacturer of the wristband, featured by a joint sparse spectrum reconstruction algorithm to remove motion-related artifacts^32^. The GSR data were simply downsampled from the original 40-Hz raw data, and the motion data were computed as the downsampled root mean square of the 20-Hz three-axis raw acceleration data to reflect the overall motion intensity. Note that the minimal output value of heart rate value from the device was 40. The value of 40 was obtained when 1) no valid heart rate could be computed due to unreliable PPG signal acquisition; 2) the computed heart rate value was less than 40. In addition, the minimal output value of GSR from the device was 0.00624 μS, indicating high skin impedance that could be caused by relatively loose skin contact. As these data could be meaningful (although likely to be missing values), these values were kept in the data file and the users are suggested to interpret them with caution. The organization of the contents in [Physiol_Rec].CSV is shown in Table 4.

**Table 4:** Organization of the contents for physiological data in [Physiol_Rec].CSV.

Column	Content	Unit	Sampling rate
1	Heart rate	bpm	1 Hz
2	Motion intensity (R.M.S of accelerations)	m/s^2^	1 Hz
3	GSR	μS	1 Hz
4	Device battery information	%	1 Hz
5	Recording time (yyyy/mm/dd HH:MM:SS)	s	1 Hz

In addition, there were three more CSV files in the same directory with post-fixes of “_ACC”, “_GSR” and “_PPG” to [Physiol_Rec], with raw data at the original sampling rate for the three-axis acceleration, GSR and PPG, respectively. The raw data were intended for experienced researchers to further explorations. The organization of the contents were explained in Tables 5, 6 and 7.

**Table 5:** Organization of the contents in [Physiol_Rec]_ACC.CSV.

Column	Content	Unit	Sampling rate
1	Acceleration in x axis	m/s^2^	20 Hz
2	Acceleration in y axis	m/s^2^	20 Hz
3	Acceleration in z axis	m/s^2^	20 Hz
4	Recording time (yyyy/mm/dd HH:MM:SS)	s	20 Hz

**Table 6:** Organization of the contents in [Physiol_Rec]_GSR.CSV.

Column	Content	Unit	Sampling rate
1	GSR responses	μS	40 Hz
2	Recording time (yyyy/mm/dd HH:MM:SS)	s	40 Hz

**Table 7:** Organization of the contents in [Physiol_Rec]_PPG.CSV.

Column	Content	Unit	Sampling rate
1	PPG signals	a.u.	20 Hz
2	Recording time (yyyy/mm/dd HH:MM:SS)	s	20 Hz

### Scripts

Preprocessing and primary analysis codes (MATLAB scripts) are summarized in this directory. All the results in the technical validation section can be reproduced using these scripts. For more detailed information, please read the instruction and descriptions in the README.TXT file alongside the data at Synapse^39^.

## Technical Validation

### Psychological recordings

For the ESM data, a total of 3789 events were recorded, with an average of 26.7 ± 3.5 ($\bar{x}\pm s$) assessments per participant, corresponding to an average response rate of 88.9 ± 11.5%. For every single participant, the number of reported events ranged from 12 to 30, and the response rate varied from 40% to 100%. While for the DRM data, a total of 3901 DRM events were recorded, corresponding to 27.7 ± 5.9 events per participant (ranging from 0 to 41 events).

**Fig. 2:**
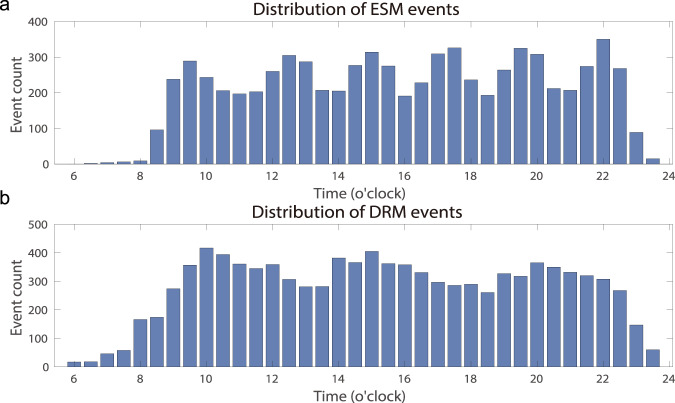
The time distribution of the ESM and DRM events. Each bar represents the number of events within a 30-minute time bin.

**Fig. 3:**
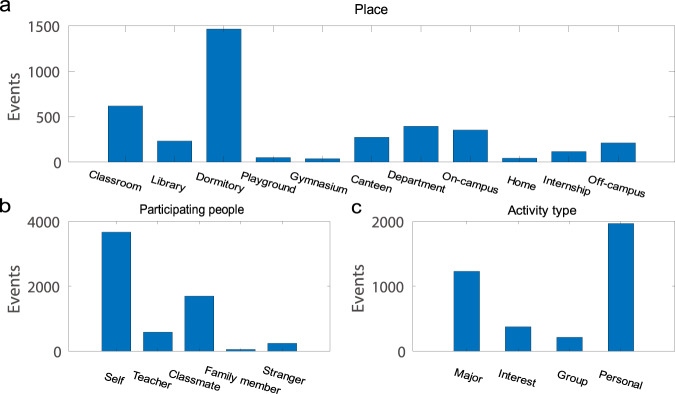
The basic event information of the ESM records.

**Fig. 4:**
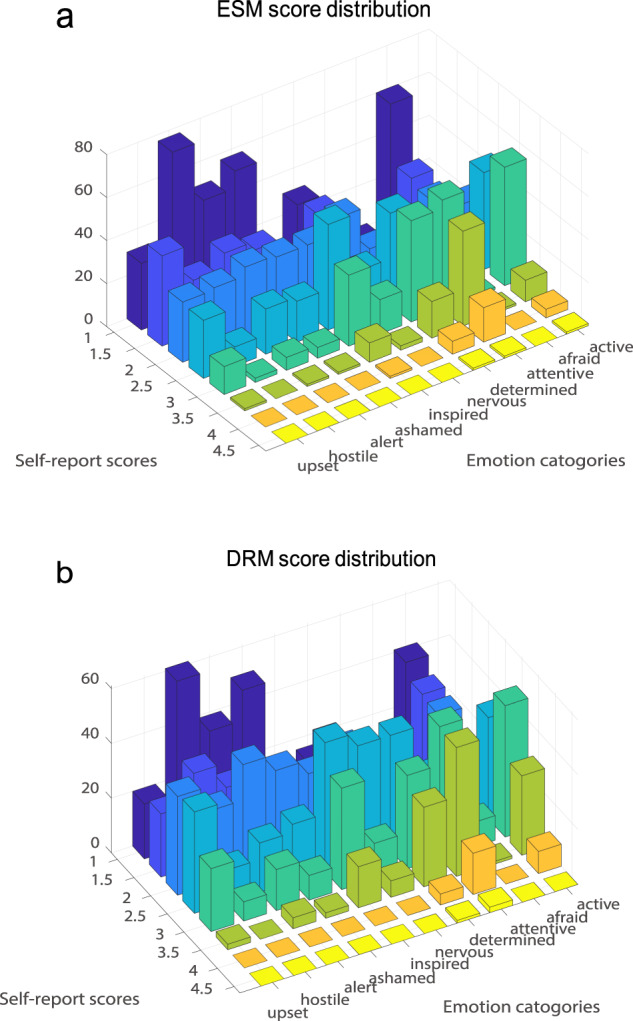
Summary of the participants’ ratings of their momentary emotional states.

Figure 2 shows the time distributions of the collected ESM and DRM events across all participants. The events were relatively evenly distributed during the day time (>100 events for most of the 30-minute time bins), with peaks at 9:00 a.m., 12:00 p.m., 3:00 p.m., 5:00 p.m., 8:00 p.m., and 10:00 p.m. for ESM events and peaks at 10:00 a.m., 3:00 p.m., and 8:00 p.m. for DRM events. Figure 3 further illustrates the basic event information of the ESM events. The most frequently chosen options for the event place are dormitory, classroom, and department building. Self and classmates are the most common participants of the ESM events. Handling non-academic personal affairs accounted for 51.9% of the sampled events. Note that 83 DRM events (2.12%) are not shown in Fig. 2 as they were within 0:00 a.m. and 6:00 a.m. The participants’ ratings of their momentary emotional states are summarized in Fig. 4.

**Fig. 5:**
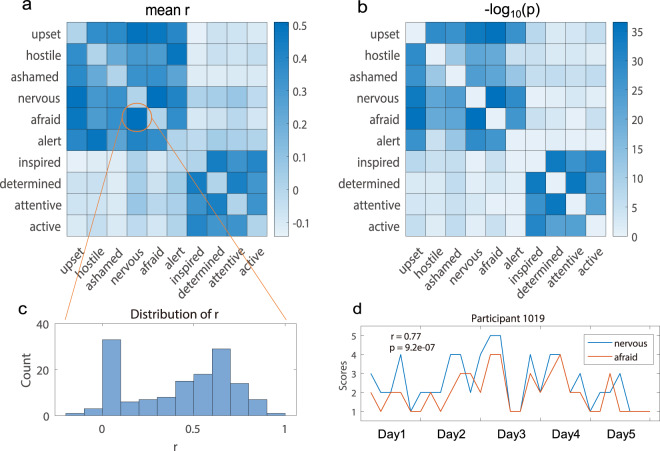
(**a**) Averages of the within-participant bivariate correlations among the ten emotion categories based on the ESM data. (**b**) The significance level as represented by -log_10_ transformation of the p-values about the significant differences between the distribution of participant-level correlation values and zero. (**c**) The distribution of the r-values calculated between nervous and afraid. (**d**) An illustration of the ESM data of one representative participant (ID: 1019).

**Fig. 6:**
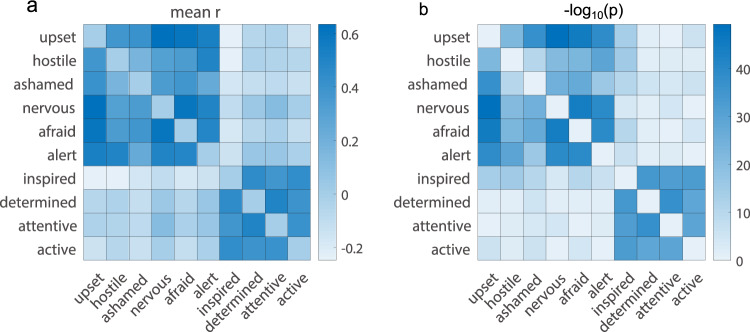
(**a**) Averages of the within-participant bivariate correlations among the ten emotion categories based on the DRM data. (**b**) The significance level as represented by -log_10_ transformation of the p-values about the significant differences between the distribution of participant-level correlation values and zero.

The bivariate correlations among the ten emotion categories are shown in Fig. 5 (for ESM) and Fig. 6 (for DRM). The displayed correlation r-values are the averages of the within-participant Pearson correlations across all participants (maximal 30 ESM events per participant for the calculation). The displayed p-values (-log_10_(p) transformed) are from one-sample t-tests about whether the averages of the within-participant correlations of all participants were significantly different from zero. The correlation coefficients were transformed into z-values before performing the t-tests. Top significant correlations were observed for the pairs of nervous-afraid ($\bar{{\rm{r}}}$ = 0.51, p = 3.3 × 10^−37^), upset-afraid (r = 0.47, p = 2.6 × 10^−36^), determined-attentive (r = 0.43, p = 4.4 × 10^−35^), etc. The bivariate correlation between nervous and afraid from one representative participant is illustrated in Fig. 5c. A similar pattern can be observed in the DRM bivariate correlations matrix as well (Fig. 6). These observed similarities between the ESM-based and DRM-based results are in good agreement with the previous studies^40–43^.

**Fig. 7:**
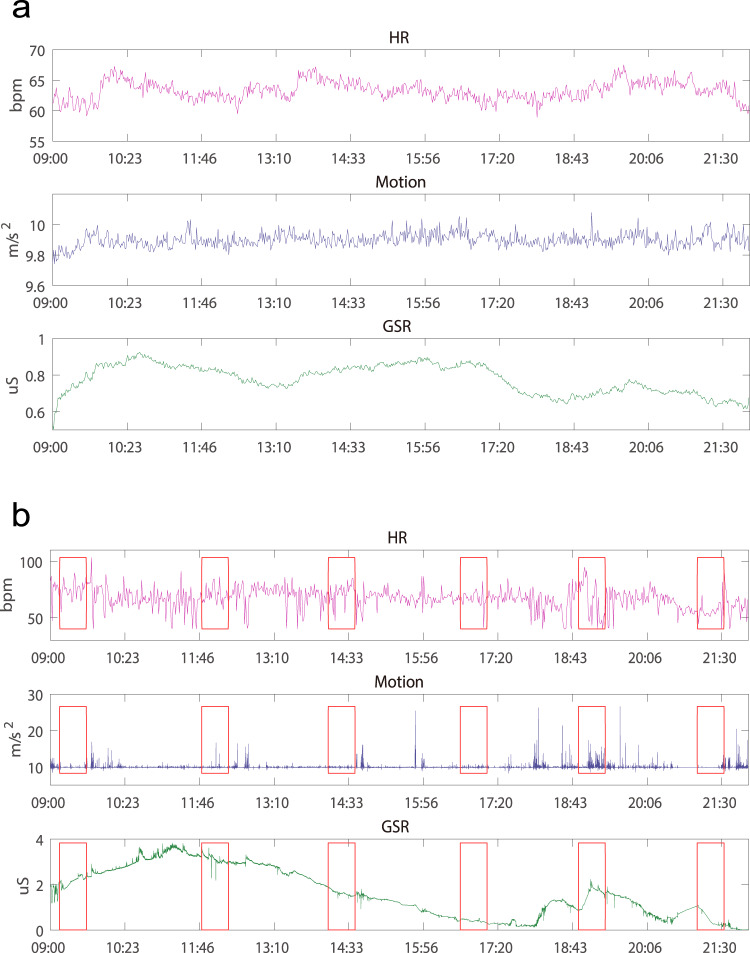
(**a**) The grand-average physiological recording over all days of all participants. (**b**) Physiological recording from a one-day period of a single participant (ID 1001, 2019/11/24). The red rectangles represent the timings of the ESM events on that day.

32.8 hours of physiological recordings per participant were obtained, summing up to 2883.4 hours recording in total. The grand average over all days of all participants is shown in Fig. 7a. While the GSR signals tended to get higher in the middle day time, especially around 10:30 and 16:00, these signals, in general, remained relatively stable across the day time from the grand average perspective. However, these signals tended to vary substantially at the single-participant and single-day level, as the example given in Fig. 7b,which is from a one-day period of a single participant (ID 1001, 2019/11/24).

The possible link between the psychological recordings (e.g., ESM records) and the physiological recordings were then explored. To this end, the physiological recordings during the 30-minute ESM periods were extracted. After excluding the ESM samples without available physiological data, a total of 1475 physiological data segments were retained. We took further procedures to minimize the influence of artefacts as they were commonly observed in daily settings. Briefly speaking, the NoiseTools toolbox^62^ was employed to identify noise through the deviation between signal and polynomial fitting results and fix the neurophysiological data if enough valid data and signal-noise ratio were detected. The details of the preprocessing method can be found in the EXTRACTFEATURES.M scripts. With the artefact-corrected data, we extracted the median value and interquartile range from all 30-minute neurophysiological data during the ESM period as indexes to preliminarily explore the neurophysiological representation of emotions in daily settings. The median and interquartile are considered to reflect the tonic level and fluctuation of corresponding neurophysiological signals over the ESM period.

**Fig. 8:**
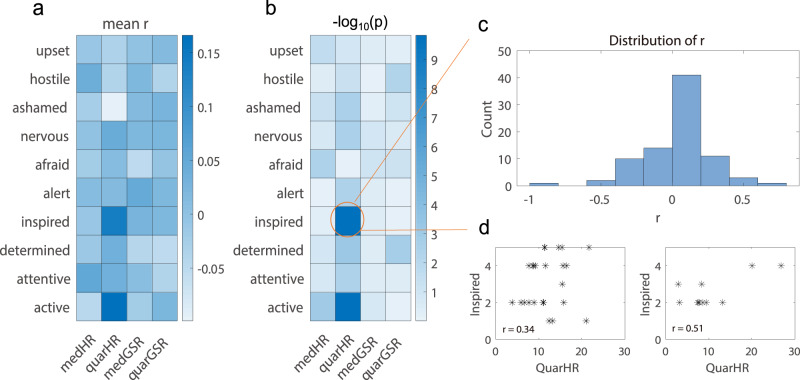
(**a**) Averages of the within-participant bivariate correlations between the neurophysiological indexes and self-reported emotion state ratings from the ESM records. medHR, quarHR, medGSR, quarGSR represent median of HR, interquartile of HR, median of low frequency GSR, interquartile of low frequency GSR, respectively. (**b**) The significance level as represented by -log_10_ transformation of the p-values about the significant differences between the distribution of participant-level correlation values and zero. (**c**) The distribution of the r-values calculated between inspired and the interquartile range of heart rate. (**d**) Two scatter plots from two representative participants (r = 0.34 and r = 0.51, respectively). Each asterisk represents the data from one ESM event period.

Averages of the within-participant bivariate correlations between these neurophysiological indexes and self-reported emotion state ratings from the ESM records are shown in Fig. 8a,b. The most significant correlations were observed between interquartile of HR and active ($\bar{{\rm{r}}}$ = 0.17, p = 5.9 × 10^−5^), interquartile of HR and inspired ($\bar{{\rm{r}}}$ = 0.14, p = 5.8 × 10^−5^), etc. The correlations between the interquartile range of HR and ratings of inspired are illustrated in details as one example (Fig. 8c,d): the distribution of the within-participant correlations suggest that most of the participants have a positive correlation; in other words, when the participants felt more inspired, there would be a greater fluctuation of their 1-Hz heart rates within this period.

## Data Availability

The data preprocessing and validation procedures presented in the validation section were conducted in MATLAB 2019b. Feature extraction of physiological signals was performed using NoiseTools toolbox, which need be previously installed in MATLAB. The README files and the data analysis code are included in the dataset^39^. Therefore, readers can easily reproduce the experimental results with the demo code and toolbox.
